# Electron Loss and Dissociation Pathways of a Complex Dicarboxylate Dianion: EDTA^2–^

**DOI:** 10.1021/acs.jpca.4c06679

**Published:** 2024-12-11

**Authors:** Jemma A. Gibbard

**Affiliations:** Department of Chemistry, Durham University, Durham DH1 3LE, United Kingdom

## Abstract

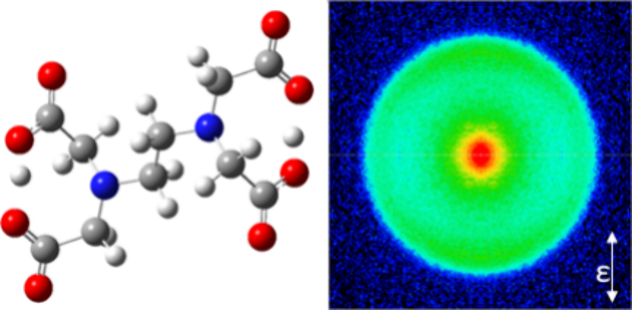

Photoelectron imaging of the doubly deprotonated ethylenediaminetetraacetic acid dianion (EDTA^2–^) at variable wavelengths indicates two electron loss pathways: direct detachment and thermionic emission from monoanions. The structure of EDTA^2–^ is also investigated by electronic structure calculations, which indicate that EDTA^2–^ has two intramolecular hydrogen bonds linking a carboxylate and carboxylic acid group at either end of the molecular backbone. The direct detachment feature in the photoelectron spectrum is very broad and provides evidence for a dissociative photodetachment, where decarboxylation occurs rapidly after electron loss. Near 0 eV kinetic energy electrons are only observed in the photoelectron spectrum of EDTA^2–^ at *h*ν = 3.49 eV (high laser fluence), providing evidence for secondary electron loss via a two–photon process, mediated by an excited state of the decarboxylated anion, and likely resulting in a cyclic neutral product.

## Introduction

The conjugate bases of ethylenediaminetetraacetic acid (EDTA, [CH_2_N(CH_2_CO_2_H)_2_]_2_) are chelating ligands which can form stable complexes with most metal cations. For example, edetate disodium, which contains the doubly deprotonated EDTA dianion (EDTA^2–^), is a medicine used to treat calcium overload, by coordinating to calcium within the body. Typically, the EDTA conjugate base coordinates to the metal center via four carboxylate groups, and two N groups (amine), to form metal-EDTA complexes with octahedral symmetry. The resulting singly and doubly charged metal complexes have been studied extensively in the solution-phase, and more recently by gas-phase ion spectroscopy, but the structure and dynamics of isolated EDTA^2–^ is still unknown.^1−5^ The most common forms of the EDTA conjugate bases, depending upon the pH of the solution, are multiply charged anions, e.g., EDTA^2–^ and EDTA^4–^.

Multiply charged anions have a distinctive electronic structure that balances long–range repulsion (electron–anion) with short-range attraction (chemical bonding), described by the repulsive Coulomb barrier (RCB).^6−12^ The prototypical multiply charged anions are the aliphatic dicarboxylate dianions, which have been extensively studied using photoelectron spectroscopy, and exhibit several interesting features.^13−16^ By increasing the length of the carbon backbone in the dicarboxylate dianions, a decrease in the minimum height of the RCB and an increase in the electron binding energy (eBE) is observed, converging to the eBE of a linear aliphatic carboxylate (∼3.2 eV).^16^ Furthermore, low energy electrons were observed in photoelectron imaging experiments, providing evidence for exotic dynamics in the form of a secondary dissociative autodetachment process (i.e., double electron loss and double decarboxylation upon the absorption of a single photon).^17^ Finally, the anisotropy of the photoelectron angular distribution (PAD) for direct detachment (nonzero electron kinetic energy (eKE)) depended strongly upon the chain length of the dicarboxylate dianion and the photon energy used, such that PADs characterized by both positive and negative anisotropy parameters (*β*_2_) were observed, despite the transition consistently having perpendicular character.^18,19^

**Figure 1:**
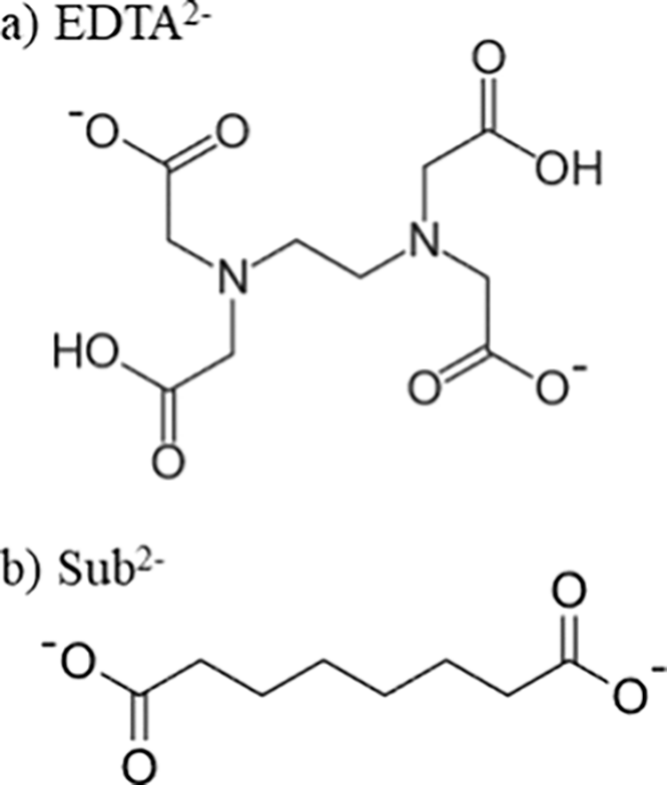
The structure of the dicarboxylate dianions (a) EDTA^2–^ and (b) Sub^2–^.

In this manuscript we study the photodetachment of a complex dicarboxylate dianion, EDTA^2–^, which contains N atoms in its molecular backbone and has additional side chains that terminate in carboxylic acid groups, using photoelectron spectroscopy for the first time. This additional complexity allows us to study the effect of heteroatoms, as well as the potential for additional screening of the negative charges by side chains, intramolecular bonding and cyclization reactions, on the structure and dynamics of a dicarboxylate dianion. For comparison I also studied the doubly deprotonated suberic acid dianion (CO_2_^–^(CH_2_)_6_CO_2_^–^, Sub^2–^).^16,17^ Both Sub^2–^ and EDTA^2–^ have six atoms in the molecular backbone separating two negativly charged carboxylate groups, but the backbone of EDTA^2–^ contains C and N atoms whereas Sub^2–^ contains just C atoms. The chemical structures of EDTA^2–^ and Sub^2–^ are shown in Figure 1.

## Methods

Photoelectron spectroscopy was performed using an apparatus which will be summarized here, as it has been described in detail elsewhere.^20,21^ EDTA^2–^ and Sub^2–^ were produced via electrospray ionization of a 1 mM solution of EDTA or suberic acid in methanol, where ammonia was used to deprotonate the parent acid. Anions enter the apparatus via a capillary, are guided and trapped using a series of radiofrequency guides before acceleration to 2.2 kV using a Wiley–McLaren time–of–flight mass spectrometer.^22^ The mass selected ion packet of EDTA^2–^ was overlapped with the nanosecond output from either a Nd:YAG pumped optical parametric oscillator (*h*ν = 4.0 eV, 3.75 and 3.64 eV) or the harmonics of a Nd:YAG laser (*h*ν = 4.66 and 3.49 eV). Electrons ejected from the dianions are recorded on a position sensitive detector, consisting of microchannel plates and a phosphor screen, using velocity map imaging. The resulting photoelectron image, which is accumulated for a minimum of 5000 shots, is processed using the polar onion peeling algorithm to result in the photoelectron spectrum, which can be presented in terms of eKE or electron binding energy (eBE) using the photon energy (*h*ν = eKE + eBE), and the PAD.^23^ The PAD is characterized in terms of an anisotropy parameter (*β*_2_) which runs from −1 (perpendicular transition) to +2 (parallel transition).^24−26^ Laser power dependence measurements were also performed to determine if any of the electron loss pathways were multiple photon processes. The photoelectron imaging spectrometer has an energy resolution of ∼5%, as determined from the well-known photoelectron spectrum of I^–^.

Electronic structure calculations are used to aid the interpretation of the experimental data for EDTA^2–^. Geometry optimizations were performed, and the minima confirmed via vibrational analysis. All energies were zero–point energy corrected. Ground state calculations used density functional theory (DFT) within Gaussian 16, at the CAM–B3LYP level of theory, with the aug–cc–pVTZ basis set.^27−29^ Excited state calculations were performed using time–dependent DFT and the Tamm–Dancoff approximation, using both B3LYP/aug–cc–pVTZ and B3LYP/6-311++**, which gave consistent results.^28,30−32^ Furthermore, I calculated the Franck–Condon envelope for photodetachment of EDTA^2–^ using the Newton-X 2.0 package, with CAM–B3LYP/aug–cc–pVTZ.^28,29,33^ A nuclear ensemble approach was utilized whereby 250 geometries around the optimized dianion ground state geometry were randomly sampled at 300 K, before the ground state of the monoanion (D_0_) was computed, and the overlap determined by Koopman’s approximation for each geometry. The final spectrum was produced by applying a Lorentzian with width 0.15 eV to each transition, to account for the spectral broadening arising from the resolution of the photoelectron imaging spectrometer (5% of 3 eV).

## Results

### a) Photoelectron Imaging of EDTA^2–^

**Figure 2:**
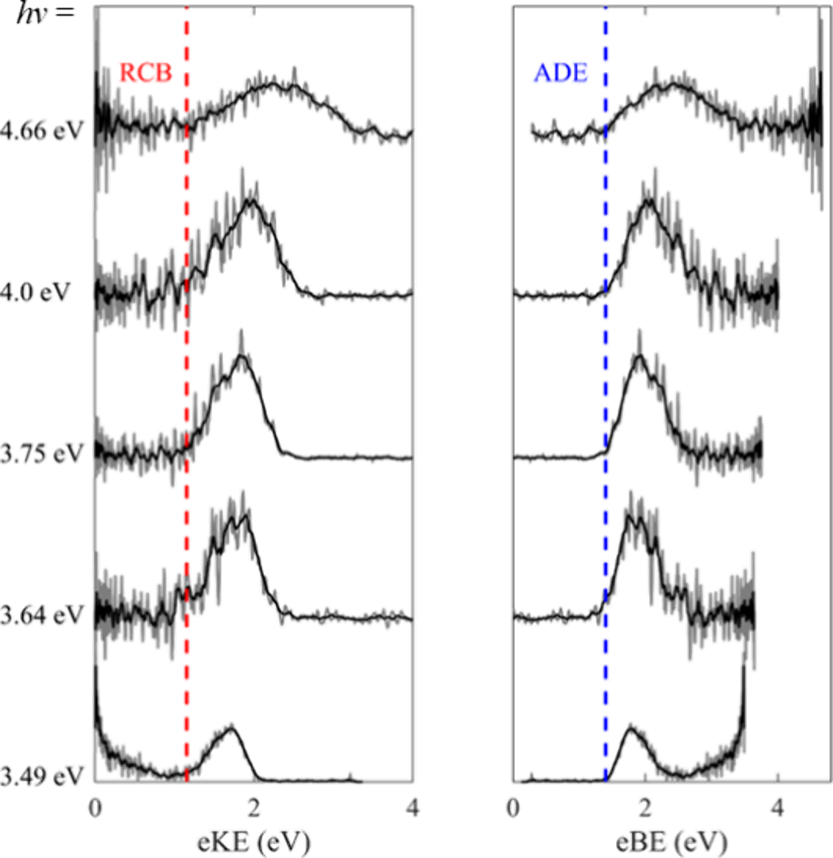
Photoelectron spectra of EDTA^2–^ at a range of wavelengths on an eKE (left-hand side) and an eBE (right-hand side) scale. The raw data is plotted in gray, while a five-point moving average is shown in black. The minimum height of the RCB (red) and ADE (blue) are highlighted.

The photoelectron spectra of EDTA^2–^ recorded at different photon energies and reported on both an eKE and eBE scale are shown in Figure 2. Two features are seen in the spectra: first, a band with a spectral onset at eBE = 1.4 eV ± 0.1 eV and broadening out with photon energy, and second, a narrow feature of near 0 eV eKE electrons, on top of a broader band of electrons with eBE < ∼ 1 eV, present in the *h*ν = 3.49 eV spectrum.

The first feature is consistent with direct detachment over the RCB and allows us to extract an adiabatic detachment energy (ADE) for EDTA^2–^, ADE ≈ 1.4 eV (highlighted by the dashed blue line in Figure 2). As the photon energy increases the bandwidth broadens to ∼2 eV at *h*ν = 4.66 eV, as more of the Franck–Condon envelope becomes energetically accessible (i.e., *hv* ≫ ADE + RCB). Such a broad spectral profile is suggestive of a large geometry change upon photodetachment (i.e., between the dianion and anion: EDTA^2–^ vs. EDTA^–^). From the highest photon energy spectrum, *hv* = 4.66 eV, we can also extract a vertical detachment energy (VDE), VDE = 2.4 eV ± 0.1 eV. In the photoelectron spectra of multiply charged anions the presence of an RCB cuts off the lowest energy photoelectrons, as these electrons have insufficient kinetic energy to pass over the barrier, allowing us to estimate the RCB’s minimum height.^24,34^ In this case the broad Franck–Condon envelope means that we see the effect of the RCB on the direct detachment channel at all photon energies studied, which allows us to estimate the RCB’s minimum height, RCB = 1.2 eV ± 0.2 eV (red dashed line in Figure 2). In many other photoelectron spectra of dianions, the spectral cutoff due to the RCB is observed to be a sharp edge.^25,35^ For EDTA^2–^, this is not the case, which may be reflective of the initial ion internal energy (ions are thermalizd at ∼300 K in the ion trap) and/or the relative delocalization of the charges across the carboxylate moieties.

**Figure 3:**
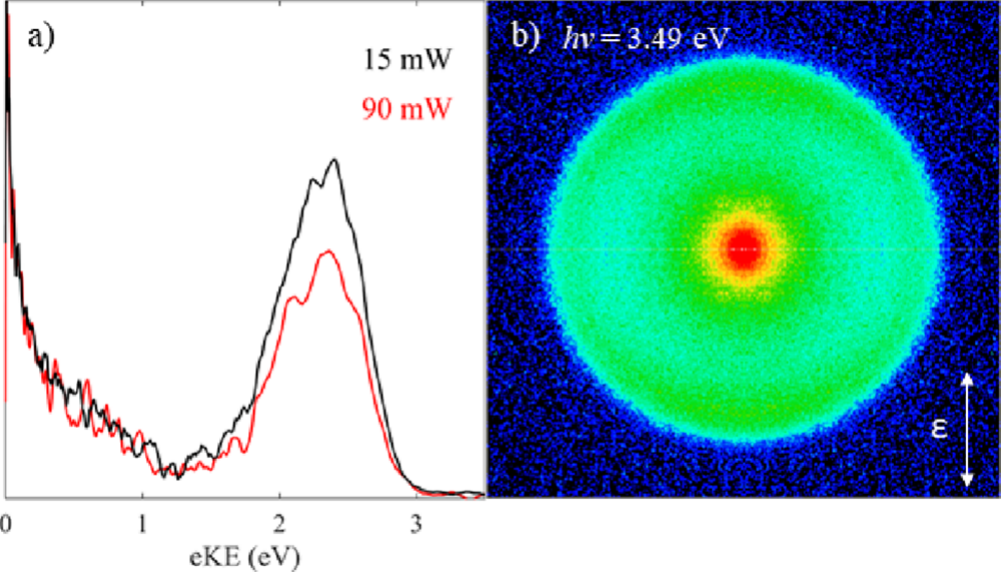
(a) Photoelectron spectra of EDTA^2–^ recorded at different average laser powers and normalized to the peak of the low eKE electrons for clarity, and (b) photoelectron image of EDTA^2–^ showing the positive anisotropy of the direct detachment channel (*β*_2_ ≈ 0.5). All spectra are recorded at *h*ν = 3.49 eV.

The narrow band of near 0 eV eKE electrons in the second feature is characteristic of thermionic emission, where electrons are “boiled” from hot parent anions, while the broader, low intensity band of eKE < 1 eV electrons is likely to be attributable to vibrational autodetachment.^36−38^ Given that the RCB stops low kinetic energy electrons arising from the photodetachment of dianions directly, these electrons must arise from thermionic emission of an anionic photoproduct instead. The source of the low eKE electrons could be EDTA^–^ or an anionic fragment. This feature is clearly present in the *h*ν = 3.49 eV spectrum, but not at the other photon energies studied providing evidence for an excited state process. Laser power dependent measurements at *h*ν = 3.49 eV are shown in Figure 3a). Relatively large low eKE signals are observed in all the spectra, but there is a small increase in the relative proportion of low eKE electrons compared to the one-photon direct detachment channel seen with increasing laser fluence, providing evidence for a multiple photon process. The identification of this channel will be discussed in detail below.

Finally, the photoelectron image of EDTA^2–^ recorded at 3.49 eV is shown in Figure 3b. In addition to the photoelectron spectra (Figure 2), photoelectron imaging provides information on the PAD. The thermionic emission (at small radii correlating with low eKE) is isotropic, as expected for a statistical process. In contrast, the direct detachment channel (at large radii correlating with high eKE) has a positive anisotropy characterized by *β*_2_ = 0.5 ± 0.2, indicating a parallel transition. At *h*ν = 4.66 eV the anisotropy of the direct detachment channel is reduced and characterized by *β*_2_ = 0.2 ± 0.2.

### b) Photoelectron Imaging of Sub^2–^

**Figure 4:**
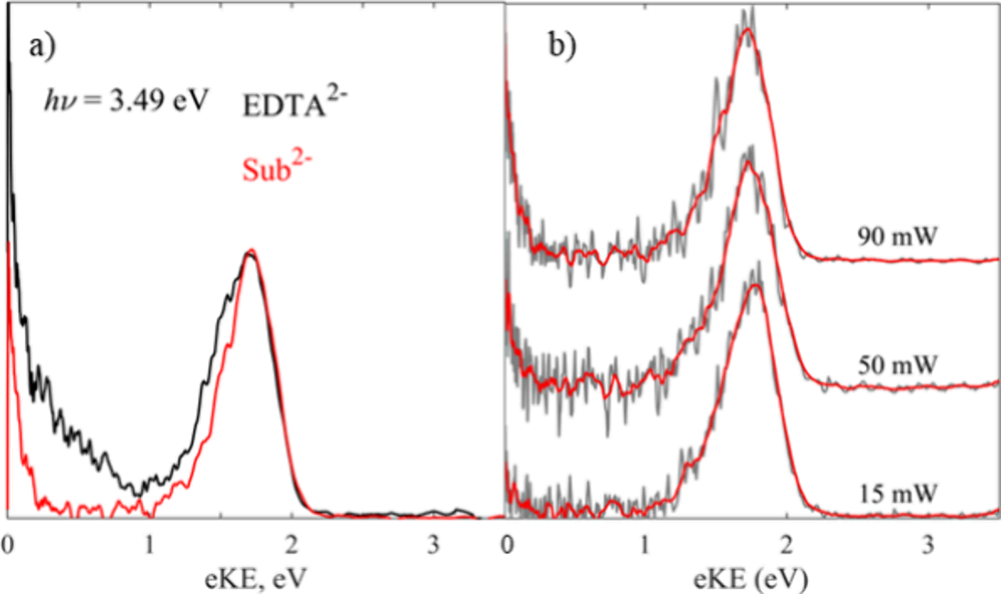
Photoelectron spectrum of (a) EDTA^2–^ (black) and Sub^2–^ (red) and (b) the Sub^2–^ photoelectron spectra recorded at various average laser powers. All spectra are recorded at *h*ν = 3.49 eV, normalized to the intensity of the direct detachment feature and presented on an eKE scale.

For contrast to EDTA^2–^, we also studied the simplest dicarboxylate dianion of similar size, Sub^2–^, and the photoelectron spectra for the two molecules recorded at *h*ν = 3.49 eV are shown in Figure 4a. The photoelectron spectra for EDTA^2–^ and Sub^2–^ are very similar, containing both a direct detachment feature and some thermionic emission, but much less evidence of vibrational autodetachment for Sub^2–^ (eKE < 1 eV). The ADE is similar for both dianions, while the RCB is slightly higher for Sub^2–^ than EDTA^2–^, based on the low eKE cutoff of electron signal. Furthermore, we observed that the proportion of thermionic emission, compared to direct detachment, increases for Sub^2–^ at *h*ν = 3.49 eV with increasing laser power (Figure 4b). This suggests that the thermionic emission in the photoelectron spectrum of Sub^2–^ arises from a multiple photon process, in contrast to the previously published assignment of a one-photon dissociative secondary autodetachment process.^17^

### c) Electronic Structure Calculations

**Figure 5:**
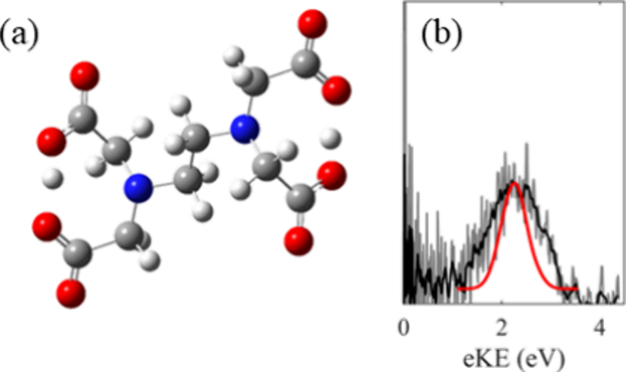
(a) Optimized geometry for EDTA^2–^, showing the two intramolecular hydrogen bonds between the carboxylate and carboxylic acid groups at either end. (b) The *h*ν = 4.66 eV photoelectron spectrum of EDTA^2–^ (black and gray from Figure 2) with the computed Franck–Condon envelope shown in red.

Electronic structure calculations were performed to determine the geometry of EDTA^2–^ and its corresponding anion, plus the relative energetics of the dianion and any potential photoproducts. The optimized ground state structure of EDTA^2–^, shown in Figure 5a, is stabilized by two intramolecular hydrogen bonds. The two negative charges are localized on single carboxylate groups at either end of the molecule, to minimize the electrostatic repulsion, and each carboxylate group is stabilized by hydrogen bonding from the neighboring carboxylic acid group, leading to a shared proton. Localizing the negative charges on carboxylate groups connected to the same substituted amine group results in an isomer which is calculated to be 2.13 eV higher in energy than the ground state, and therefore highly unlikely to be present in our anion beam. The calculated VDE of EDTA^2–^ is 2.45 eV in Table 1 and matches well with the value extracted from the *h*ν = 4.66 eV spectrum in Figure 2 (VDE = 2.4 ± 0.1 eV).

**Table 1:** Calculated Relative Energetics of EDTA^2–^, and Possible Photoproducts, Using CAM–B3LYP and Aug–cc–pVTZ^a^

**species**	**relative energy**	**VDE/(VEE)**
EDTA^2–^ (S_0_)	0	2.45
[EDTA–CO_2_]^−^ (D_0_) + CO_2_ + e^–^	1.26	4.73
c(EDTA–CO_2_)^−^ (D_0_) + CO_2_ + e^–^	2.04	2.36
c(EDTA–2CO_2_) (S_0_) + 2CO_2_+ 2e^–^	2.07	
c(EDTA–CO_2_) (S_0_) + CO_2_ + 2e^–^	3.05	
c(EDTA–2CO_2_)^−^ (D_0_) + 2CO_2_ + e^–^	3.08	0.13
[EDTA–CO_2_]^−^ (D_1_) + e^–^	4.45	(3.19)

a Calculated VDE and vertical excitation energies (VEE), where relevant. All energies are in eV.

Performing a geometry optimization on EDTA^–^ leads to CO_2_ loss, suggesting that decarboxylation occurs rapidly following electron loss from EDTA^2–^ to form a decarboxylated anion ([EDTA–CO_2_]^−^) and that the D_0_ state of EDTA^–^ is likely to be repulsive along this coordinate. At the anionic end of [EDTA–CO_2_]^−^ the shared proton present in the dianion (Figure 5a) is maintained, while at the radical end, where decarboxylation has occurred, the previously shared proton is localized onto a carboxylic acid group. In order to confirm that EDTA^–^ is unstable with respect to decarboxylation, we attempted to record the fragment mass spectrum using a reflectron secondary mass spectrometer, but this was unsuccessful.^39^ Nevertheless, evidence for photodetachment to a dissociative state can be found in the broad spectrum observed for direct detachment from EDTA^2–^ and to gain further insight, the Franck–Condon envelope for photodetachment from EDTA^2–^ was computed using a nuclear ensemble approach. The computed spectrum (red, Figure 5b) was found to be broad and to match reasonably well with the experimental photoelectron spectrum of EDTA^2–^ recorded at *h*ν = 4.66 eV (black, Figure 5b). The difference between the spectrum and simulation may indicate that the vibrational temperature of the dianions is larger than 300 K. It should be noted that no attempt is made in the simulations to account for the effect of the RCB on the photoelectron spectrum.

**Figure 6:**
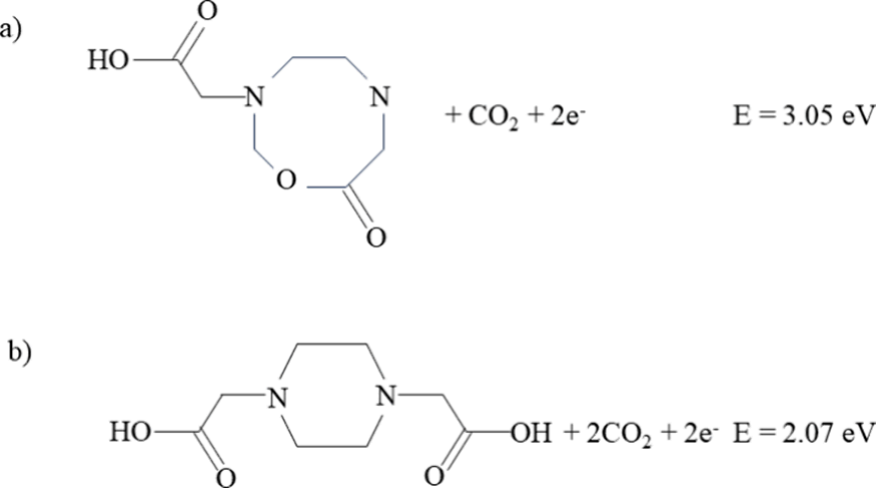
Dissociation asymptotes for the secondary photodetachment process, (a) c(EDTA-CO_2_) + CO_2_ + 2e^–^ and (b) c(EDTA-2CO_2_) + 2CO_2_ + 2e^–^, with energies relative to EDTA^2–^ (S_0_).

To aid assignment of the near 0 eV eKE electrons, we also considered the structure and energetics of the likely primary photoproduct of photodetachment of EDTA^2–^: [EDTA–CO_2_]^−^. The VDE of [EDTA–CO_2_]^−^ was computed to be VDE = 4.73 eV, and the decarboxylated anion was also found to have a relatively low-lying electronically excited state, with a vertical excitation energy (VEE) computed to be VEE = 3.19 eV. Calculations indicated that electron loss from [EDTA–CO_2_]^−^ can lead to the formation of the relatively stable cyclic neutral products: c(EDTA–CO_2_) or c(EDTA–2CO_2_), which is formed by the loss of an additional CO_2_ group. These possible product channels are shown in Figure 6, with the energies given relative to EDTA^2–^. The six membered cyclic product c(EDTA-2CO_2_) has a negative electron affinity EA = −1.01 eV, indicating that the anion is unstable with respect to electron loss, whereas the eight membered cyclic product c(EDTA-CO_2_) has a positive electron affinity (EA = 1.01 eV), indicating a bound anion. The calculated relative energetics of EDTA^2–^ and related species are shown in Table 1.

## Discussion

First, we will consider the origin of the two features in the photoelectron spectra of EDTA^2–^ in the context of the electronic structure calculations, before comparing the data to the simple aliphatic dicarboxylate dianions. The direct detachment process observed in Figure 2, is likely to be a dissociative photodetachment, where the loss of the electron is rapidly followed by CO_2_ loss via a one–photon process. Dissociative photodetachment is commonly observed for carboxyl radicals formed via photodetachment, and has been seen in dianions before.^39−43^ The dissociation is likely to occur on a repulsive region of the potential energy surface as calculations indicate that the bond angles around the N atom in the dianion are smaller, i.e. more pyramidal, than in the decarboxylated anion [EDTA–CO_2_]^−^ (D_0_), such that upon photodetachment an anion is formed in an unfavorable geometry.^40^ It is likely that this strained geometry is favored in the dianion in order to accommodate the intramolecular hydrogen bond, which stabilizes the negative charge. The weakening of the stabilizing hydrogen bond upon electron loss, plus the formation of an anion in a unfavorable geometry, is likely to provide the driving force for a repulsive dissociation, as has previously been observed for the dissociative photodetachment of other hydrogen-bond stabilized carboxylates (e.g., C_2_O_4_H^–^).^40^

Ultimately the photodetachment to the repulsive and dissociative EDTA^–^ (D_0_) electronic state will lead to a broad Franck–Condon envelope, as observed in the very broad direct detachment band seen in the photoelectron spectra (Figure 1), particularly at the highest photon energies. Given that photodetachment leads to a repulsive state, we cannot calculate an ADE for EDTA^2–^, however, the energy of the dissociation asymptote [EDTA–CO_2_]^−^ + CO_2_ + e^–^ lies 1.26 eV above the dianion, which is similar to the experimental ADE of 1.4 eV, and broadly consistent with our conclusions.

The PAD for direct detachment of EDTA^2–^ becomes more isotropic as the photon energy is increased, being characterized by *β*_2_ ∼ 0.5 at *h*ν = 3.49 eV and *β*_2_ ∼ 0.2 at *h*ν = 4.66 eV. Typically, photodetachment from a carboxylate group in a monoanion would be expected to yield a negative *β*_2_, characteristic of a perpendicular transition. However, in multiply charged anions the RCB effects the ejection pathway of the outgoing electrons, and therefore the PAD.^19,25,26,44,45^ In this case at lower photon energies (*h*ν = 3.49 eV) the outgoing electron has low eKE and is strongly confined to ejection over the lowest part of the RCB. However, at higher photon energies (*h*ν = 4.66 eV), more kinetic energy is imparted to the electron, and additional pathways for electron loss become energetically accessible leading to a more isotropic PAD. Similar changes have previously been observed for the aliphatic dicarboxylate dianions.^18,19^

The low energy electrons observed in the *h*ν = 3.49 eV spectrum arise from the thermionic emission from hot monoanions, most likely [EDTA–CO_2_]^−^, which according to the electronic structure calculations is the product of direct detachment of the dianion. The VDE of [EDTA–CO_2_]^−^ is calculated to be 4.45 eV (Table 1), which is similar to the experimental ADE of the single deprotonated EDTA monoanion (ADE = 4.8 eV), but substantially higher than the 3.49 eV photon energy, making it unlikely that thermionic emission arises from [EDTA–CO_2_]^−^ directly.^4^ However, [EDTA–CO_2_]^−^ does have an energetically accessible electronically excited state, (VEE (D_1_) = 3.19 eV) and a number of low-lying rearrangement/dissociation asymptotes corresponding to the loss of an additional electron (Table 1/Figure 6). Therefore, it seems likely that the low eKE electrons in the *h*ν = 3.49 eV spectrum arise from a near resonant excitation in [EDTA–CO_2_]^−^ from D_0_ to D_1_ with a second photon, leading to a rearrangement or dissociation, and ultimately the formation of an anion which is unstable with respect to electron loss, either because it is vibrationally excited or has a negative electron affinity. Evidence in support of this mechanism is found in the small increase in the relative proportion of low eKE electrons, compared to one-photon direct detachment, observed in the photoelectron spectra of EDTA^2–^ at *h*ν = 3.49 eV with increasing average laser power (Figure 3a). This increase is clear evidence that the production of low eKE electrons is a multiple photon process, as if both spectral features arose from one-photon processes the relative intensities would be insensitive to laser fluence. Furthermore, a small increase may be expected if the secondary step of near resonant photoexcitation of [EDTA-CO_2_]^−^, which calculations indicate leads to secondary electron loss, has a larger crosssection than the initial photodetachment of EDTA^2–^, which is reasonable as it is mediated via an excited state ([EDTA-CO_2_]^−^ (D_1_)).

**Figure 7:**
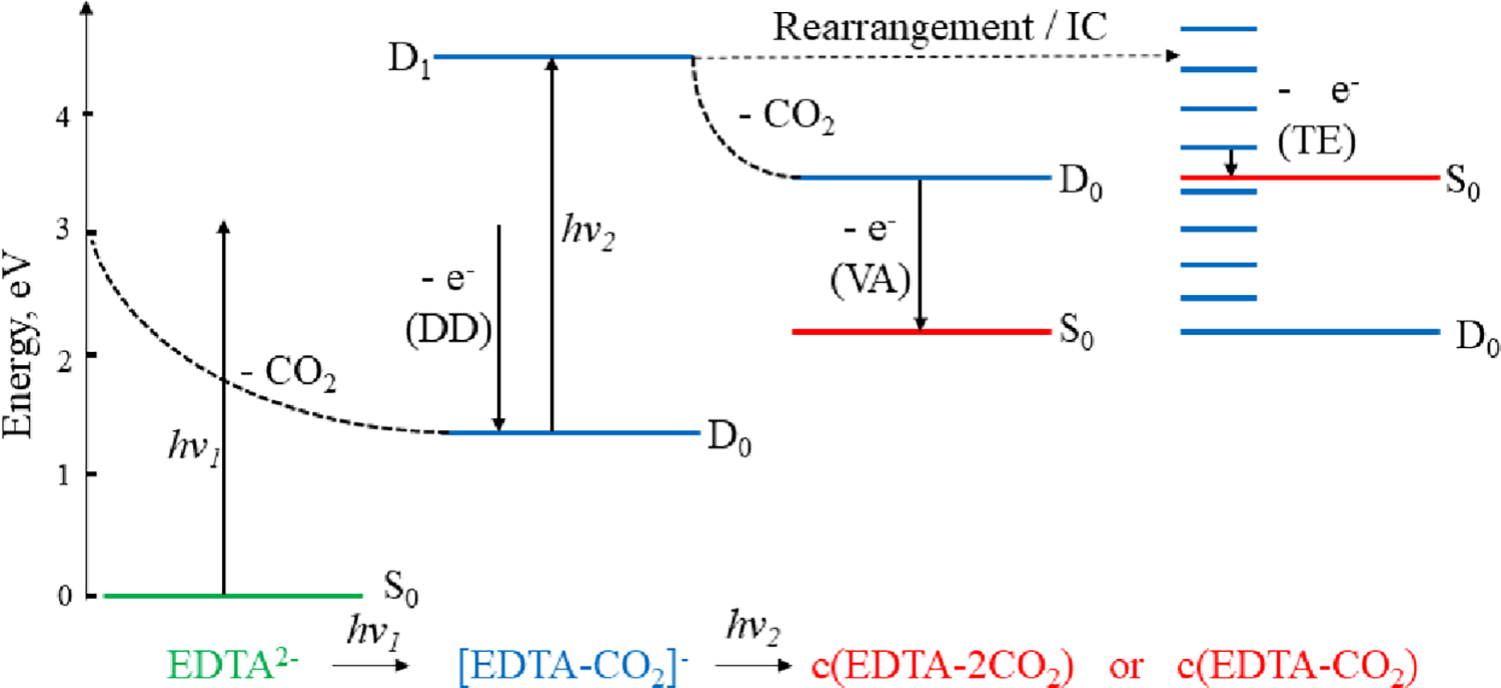
Cartoon of the electron loss processes of EDTA^2–^, showing the one-photon direct detachment pathway EDTA^2–^ → [EDTA-CO_2_]^−^ observed at all wavelengths, and the two possible secondary pathways (thermionic emission (TE) or vibrational autodetachment (VA) from monoanions) responsible for the low eKE electrons seen at *h*ν = 3.49 eV. Electronic states are colored according to the charge of the molecules: dianion (green), anion (blue) and neutral (red).

The narrow band of electrons at eKE ∼ 0 eV (Figure 1) are most likely to arise from thermionic emission of vibrationally excited c(EDTA-CO_2_)^−^, whereas the broader band of low intensity electrons (eBE < 1 eV) are probably from the vibrational autodetachment of c(EDTA-2CO_2_)^−^ (EA = −1.01 eV). Both pathways are shown in Figure 7. As we were unable to isolate EDTA-CO_2_^–^ (or EDTA^–^) using secondary mass spectrometry, it seems likely that the dissociation is either highly repulsive and therefore causes the fragments to scatter outside the reflectron, or that the dissociation is relatively slow (microseconds) which leads to a range of arrival times for the anions after the reflectron, such that clear peaks are not observable in the secondary mass spectrum. Given the broad photoelectron spectrum observed for EDTA^2–^ (Figure 2), as well as previous work on the dissociative photodetachment of carboxylates which reported repulsive decarboxylations with kinetic energy releases of up to 1.1 eV, it seems most likely that the dissociation is highly repulsive.^40,41,43^

The molecular structure of EDTA^2–^ is much more complex than Sub^2–^, but the photodetachment dynamics are very similar. For example, the ADE for Sub^2–^ is the same as for EDTA^2–^ (1.4 eV), despite amine functionalization of dicarboxylate dianions being observed to result in an increase in ADE previously, and the presence of stabilizing intramolecular hydrogen bonds in EDTA^2–^ (Figure 5a).^13^ This may be evidence that the major influence on the binding energy in a dicarboxylate dianion is the separation between charges (i.e., the length of the molecular backbone). Interestingly the height of the RCB seems to be slightly lower for EDTA^2–^ than Sub^2–^ (∼0.1 eV), based on the low eKE cutoff of the photoelectron spectra, indicating that the additional side chains from the backbone in EDTA^2–^ shield the carboxylate groups from each other somewhat. Finally, thermionic emission arising from hot anions is observed for both Sub^2–^ and EDTA^2–^, and our laser power dependent measurements suggest that this is likely to be a multiple photon process in both cases, though the mechanistic details were not studied for Sub^2–^.^17^

## Conclusions

Photoelectron imaging of EDTA^2–^ reports two distinct electron loss pathways. First, direct detachment over the RCB is observed, where loss of the electron leads to the breaking of an intramolecular hydrogen bond and subsequently decarboxylation to form [EDTA-CO_2_]^−^. The photoelectron angular distribution for direct detachment of EDTA^2–^ becomes more isotropic with increasing photon energy, as the electrons have more kinetic energy and are able to pass over higher energy regions of the RCB. Second, thermionic emission from [EDTA-CO_2_]^−^ is seen, and likely arises from near resonant excitation to the D_1_ excited state, leading to a rearrangement or dissociation, and ultimately autodetachment or statistical electron loss to form a stable cyclic product. Similarities are seen between the direct detachment channel of EDTA^2–^ and the aliphatic dicarboxylate dianion of similar backbone length, Sub^2–^, with evidence presented that both dianions produce low eKE electrons via a multiple photon process.

## Data Availability

The data which support the findings of this study are available online at https://doi.org/10.5281/zenodo.14338373.
